# Army Medical Staff

**Published:** 1875-04

**Authors:** 


For some unaccountable reason the efforts made by the profession throughout the country, in behalf of the Medical Corps of the Army, bore no fruit at the late session of Congress. The *Medical Record* of 13th March asserts that Mr. Logan, of Illinois, is responsible for the want of action. We are not familiar with the "ways that are dark" that may obtain in political circles, and do not know the particulars of the failure; but the fact remains that, notwithstanding its friends were confident that it would have passed with scarcely a dissenting voice, the bill was not even reported to either House.

This measure is in no respect partisan or political, but merely seeks to extend to the medical officers of the army those advantages of rank and pay which, by common consent, they, as a body, justly deserve. We hope that all the State medical societies at their approaching meetings will yield renewed support to the American Medical Association in its advocacy of this matter. To that end we suggest that the committees shall be so constituted as to have at least one member in each congressional district, whose particular duty it shall be to impress upon his Representative the wishes of his medical constituents, and that the chairmen shall, in like manner, see the various Senators. But action ought not to cease with the appointment of committees. Every medical man should exert his direct and constant influence in this matter, for as it now stands it appears that a single politician has antagonized the general sentiment of the faculty throughout the country.

The *American Medical Weekly* has engaged the services of one of the editors of the *Lancet* as its regular London correspondent.

